# Enabling uniform lithiation in solid-state synthesis by preventing pre-matured surface grain coarsening through grain boundary engineering†

**DOI:** 10.1039/d5sc00271k

**Published:** 2025-04-25

**Authors:** Yifan Wu, Xincan Cai, Weiyi Lin, Yingdong Deng, Qing Zhang, Haoyuan Li, Pu Yan, Guohui Zhong, Jin Xie

**Affiliations:** a School of Physical Science and Technology, ShanghaiTech University Shanghai 201210 China; b Shanghai Key Laboratory of High-resolution Electron Microscopy, ShanghaiTech University Shanghai 201210 China

## Abstract

Solid-state calcination profoundly influences the structural integrity and electrochemical performance of polycrystalline layered oxide cathode materials in lithium-ion batteries. As temperatures increase, heterogeneous phase transitions driven by solid-state diffusion can result in structural non-uniformity. In this study, we employ *operando* characterization techniques and high-resolution electron microscopy to scrutinize the inherent heterogeneity observed in the early-stage of the solid-state lithiation process and its subsequent influence on the formation and merging of resultant LiNi_0.9_Co_0.05_Mn_0.05_ (NCM90) grains on the surface of the secondary particle. We found that a conformal atomic layer deposited WO_3_ layer on the hydroxide precursor could be *in situ* lithiated to form Li_*x*_WO_*y*_ compounds, which are stable and non-dissolvable at the grain boundaries, further acting as a segregation layer to prevent the merging of grains during the formation of a layered phase on the surface of secondary particles, which preserves the route for the uniform lithiation of the inner part of the secondary particles. These investigations shed light on the effect of solid-state reaction heterogeneity and present a novel methodology for mitigating the persistent challenge by grain boundary engineering.

## Abstract 2

This work reported a novel method to enhance the solid-state calcination homogeneity of LiNi_0.9_Co_0.05_Mn_0.05_O_2_ by depositing a WO_3_ layer on precursor surface *via* atomic layer deposition, obtaining a high-performance nickel-rich layered cathode.

## Graphical abstract

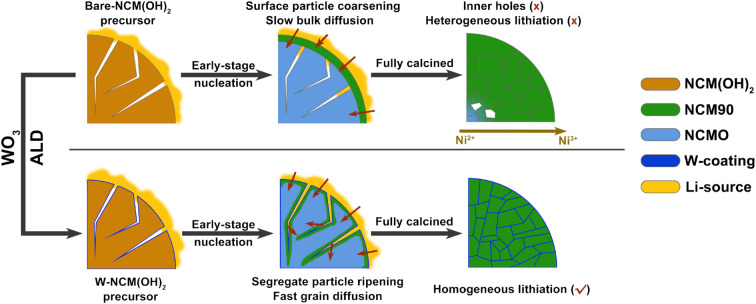

## Introduction

Solid-state reactions represent a foundational and extensively utilized synthetic method for manufacturing inorganic solid materials, particularly metal oxide ceramics. In a typical solid-state synthesis, multiple solid compounds are mixed and heated at high temperature to form new chemicals through heterogeneous reactions. Increased mixing and reaction temperatures are preferred to facilitate atomic diffusion, promoting greater product uniformity, given that the solid-state reaction occurs primarily at the interface of the reactants, where the reaction rate is dependent on the diffusivities of atoms.^1,2^ The industrial manufacturing of the widely used Li-ion battery cathode, LiTMO_2_ (where TM denotes transition metals such as Ni, Mn or Co) layered oxides, often involves a high-temperature solid-state reaction using transition-metal hydroxide precursors, TM(OH)_2_, and a lithium source, typically LiOH or Li_2_CO_3_ as starting materials in an oxidative atmosphere.^3^ This process, commonly referred to as calcination, starts with heterogeneous phase transition at relatively low temperature.^4–7^ The outcomes of the calcination, including the final structure and morphology of the LiTMO_2_ product, are governed by the competition of mass transportation and chemical reactions, which are further influenced by factors such as the calcination atmosphere, temperature profile, and precursor particle size.^8–12^

State-of-the-art characterization techniques have been employed to probe the complicated non-equilibrium heterogeneous reaction process. Previous *in situ* XRD studies have indicated that there are several competing reactions that occur in the solid-state calcination: (1) the lithiation of the precursor to form a highly-disordered lithiated phase, (2) the rearrangement of Li and TM, and (3) the growth of the layered oxide lithiated phase.^6,13–15^ Lim and colleagues utilized nano secondary ion mass spectrometry (nano-SIMS) and *ex situ* transmission X-ray microscopy (TXM) to elucidate the relationship between lithium diffusion and heterogeneous transition metal oxidation, which is strongly correlated with the growth of the layered oxide phase.^16^ The kinetics of lithium diffusion and layered oxide phase growth was further studied using environmental transmission electron microscopy (TEM), revealing that phase transition is limited by lithium diffusivity during early-stage calcination. A dense lithiated shell formed at relatively low temperatures, which suppressed further lithium transport during later calcination stages, leading to the formation of a spatially inhomogeneous product with inner voids and a rock salt phase, where the secondary particle center lacked sufficient lithium.^17,18^

Such inhomogeneity arises from the inherent heterogeneity of solid-state reactions, where the reaction predominantly occurs at the interface between solid reactants. Park *et al.* suggested that extending the lithium diffusion period at low temperature, which alleviates the formation of a surface lithiated shell, could improve particle homogeneity.^17^ Wolfman *et al.* optimized the competition between lithiation and crystal growth by using dehydrated TM(OH)_2_ as the transition metal precursor, enabling sufficient lithium incorporation at low temperatures.^19^ Other strategies, such as increasing the sphericity of TM(OH)_2_ precursors to promote the uniformity of surface reactivity^20^ and reducing the size of the TM(OH)_2_ precursor or lithium source,^9^ have also been employed to reduce lithium diffusion heterogeneity.

While most previous studies focus primarily on balancing lithium diffusion and lithiated particle growth, the intrinsic heterogeneity in solid-state calcination requires more intricate solutions. In this work, we developed a conformal WO_3_ layer using atomic layer deposition (ALD) directly on the spherically shaped polycrystalline Ni_0.9_Co_0.05_Mn_0.05_(OH)_2_ (denoted as NCM(OH)_2_) precursor particles, effectively regulating lithium diffusion and particle coarsening at grain boundaries during subsequent solid-state calcination. The WO_3_ layer would *in situ* transform into the Li_*x*_WO_*y*_ phase (denoted as LWO) on a primary particle surface, benefiting from the insolubility of tungsten atoms in the NCM lattice, proved by X-ray photoelectron spectroscopy (XPS) and energy dispersive X-ray spectroscopy analysis by transmission electron microscopy (TEM-EDS), which would allow a deeper lithium diffusion and nucleation depth by preventing the formation of a dense lithiated shell originating from the coarsening of lithiated particles in the early stage. The LWO phase on the surface of hydroxide precursors improves the lithium-ion transportation, leading to a more uniform lithiation to form high quality NCM. The detailed lithiation steps were elucidated through *operando* high-temperature synchronous X-ray diffraction (HTXRD) and Rietveld refinement analyses. This novel modification strategy of the precursor with an ALD WO_3_ coating effectively mitigates the presence of heterogeneous structures within the final particles, as observed through cross-sectional scanning electron microscopy (SEM) and high-angular annular dark-field scanning transmission electron microscopy (HAADF-STEM).

## Results and discussion

**Fig. 1:**
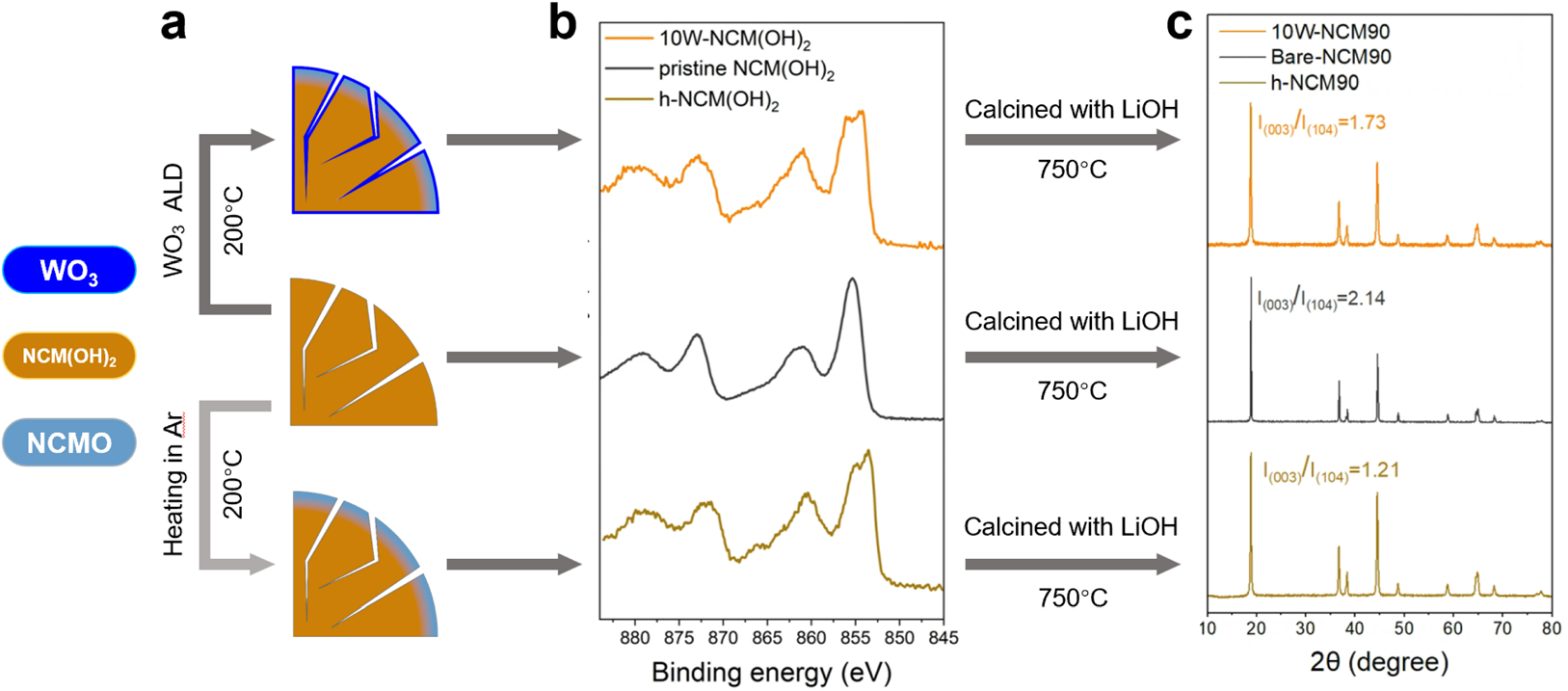
Schematics, surface properties and structural properties of hydroxide precursors (pristine NCM(OH)_2_, 10W-NCM(OH)_2_, and h-NCM(OH)_2_) and corresponding layered oxides (bare-NCM90, 10W-NCM90, and h-NCM90). (a) Schematic illustrations of the preparation process for pristine NCM(OH)_2_, 10W-NCM(OH)_2_, and h-NCM(OH)_2_. Deep blue, brown and light blue represent the WO_3_ coating, NCM(OH)_2_, and dehydrated NCMO, respectively. (b) XPS spectra for pristine NCM(OH)_2_, 10W-NCM(OH)_2_, and h-NCM(OH)_2_. (c) XRD patterns of the fully calcined samples (bare-NCM90, 10W-NCM90, and h-NCM90) at 750 °C in O_2_ with LiOH·H_2_O for 12 h.

To investigate the early-stage solid-state lithiation kinetics and the relationship between the surface reactivity of the NCM(OH)_2_ precursor and the nucleation and growth behavior of the lithiated phase at low to medium temperatures, we prepared NCM(OH)_2_ precursors using two different approaches. In the first approach, a tungsten oxide (WO_3_) layer was deposited onto the surface of powdery precursor particles *via* atomic layer deposition (ALD) at 200 °C, resulting in W-NCM(OH)_2_. In the other approach, powdery precursor particles were heated in a vacuum atmosphere at 200 °C for 3 hours (using the same heating profile as the ALD-coated samples but without exposure to ALD precursors), yielding h-NCM(OH)_2_. These samples, illustrated in Fig. 1a, were designed to simulate relatively inert (WO_3_-coated) and reactive (surface-dehydrated) surface conditions, respectively, compared to pristine NCM(OH)_2_. Detailed ALD procedures are provided in the Experimental section.

The NCM(OH)_2_ precursor exhibited a tendency to decompose during thermal treatment, as observed in the thermogravimetric analysis (TGA) (Fig. S1a and b†). Although the complete dehydration temperature of NCM(OH)_2_ in an oxygen environment is approximately 305 °C, a gradual and consistent mass loss was detected even below 200 °C. The Ni 2p XPS spectra of h-NCM(OH)_2_ (Fig. 1b) showed peak splitting between 853–856 eV, resembling the signature of NiO and distinct from Ni(OH)_2_ or NiOOH,^21,22^ indicating that the surface of NCM(OH)_2_ partially dehydrated into a rock salt phase, Ni_0.9_Co_0.05_Mn_0.05_O (denoted as NCMO). Similar structural changes were observed in the NCM(OH)_2_ precursor after 10 cycles of WO_3_ ALD coating (10W-NCM(OH)_2_; precursors after *n* cycles of ALD coating are denoted as *n*W-NCM(OH)_2_), with the surface structure transforming into a rock salt phase after ALD performed at 200 °C. X-ray diffraction (XRD) results (Fig. S1c†) further confirmed a mixed phase of NCM(OH)_2_ and NCMO in the 200 °C preheated precursor, implying that the mass loss at this stage arises from the precursor's dehydration to the rock salt phase.

The pristine (NCM(OH)_2_), pre-heated (h-NCM(OH)_2_) and ALD-coated (10W-NCM(OH)_2_) precursors were subsequently mixed with a lithium source and calcined at 750 °C in O_2_ for 12 hours to obtain bare-NCM90, h-NCM90, and 10W-NCM, respectively (see the Experimental section for synthesis details). According to XRD characterization (Fig. 1c), pristine NCM(OH)_2_ was fully converted into a layered phase, exhibiting an *I*_(003)_/*I*_(104)_ peak intensity ratio of 2.14. However, when h-NCM(OH)_2_ was used as the precursor, the *I*_(003)_/*I*_(104)_ peak ratio of the resulting h-NCM90 decreased to 1.21 under identical calcination conditions. This decrease suggests increased Li/Ni disordering or the persistence of unreacted rock salt phases,^23,24^ indicating that a reactive NCMO surface may adversely impact calcination outcomes. In contrast, when the surface of NCM(OH)_2_ was fully coated with a WO_3_ layer *via* ALD, the resulting 10W-NCM90 exhibited an *I*_(003)_/*I*_(104)_ peak intensity ratio of 1.73, which is higher than that of h-NCM90 but lower than that of bare-NCM90. The varying degree of Li/Ni mixing might be attributed to the introduction of high-valence transition metal elements like tungsten that has been reported in previous studies.^25–28^ These findings highlight the critical role of precursor surface characteristics in determining the outcome of solid-state calcination processes.

**Fig. 2:**
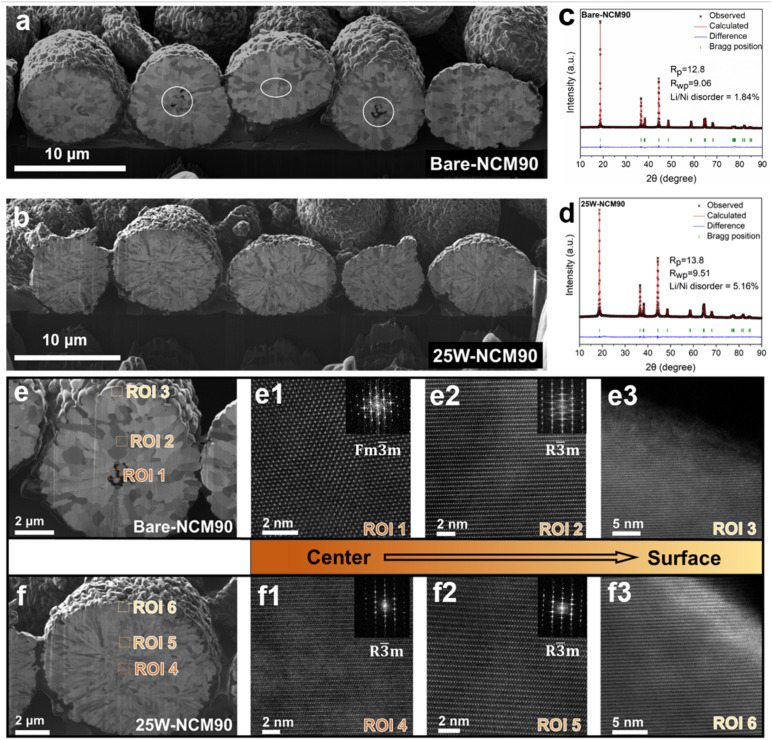
Particle cross-section morphology, XRD refinement, and lattice structure for bare-NCM90 and 25W-NCM90. (a and b) Cross-sectional SEM images of bare-NCM90 and 25W-NCM90. Internal voids and holes are marked by white circles. (c and d) XRD refinement results for bare-NCM90 and 25W-NCM90. (e) Cross-sectional SEM image of a bare-NCM90 particle; (e1–e3) HAADF-STEM images display three regions of interest (ROIs 1–3) from the center to the surface of a secondary particle in bare-NCM90. (f) Cross-sectional SEM of a 25W-NCM90 particle, (f1–f3) HAADF-STEM images display three regions of interest (ROIs 4–6) from the center to the surface of a secondary particle in 25W-NCM90. HAADF-STEM images were captured along the <100> zone axis in the *R*3̄*m* lattice or <111> zone axis in the *Fm*3̄*m* lattice.

To further investigate the origin of the decreased *I*_(003)_/*I*_(104)_ peak intensity ratio and assess lithiation uniformity within secondary particles, cross-sectional SEM and HAADF-STEM analyses were performed on bare-NCM90 and 25W-NCM90 (Fig. 2). These techniques examined the structural uniformity from the center to the surface of secondary particles to elucidate the effects of heterogeneous solid-state reactions. In bare-NCM90 (Fig. 2a), primary particles exhibit equiaxed sizes with voids concentrated near the center of secondary particles. The primary particles adjacent to these voids were significantly smaller than those closer to the surface, likely due to inhibited nucleation and grain growth of the layered phase. Considering the slow heating profile and relatively facile oxygen transport, we hypothesize that the limited lithium supply at the particle center caused the delayed lithiation. In contrast, in 25W-NCM90 (Fig. 2b), primary particles displayed a uniform rod-like morphology from the center to the surface, with no internal voids or significant size reduction observed near the centers of the secondary particles.

HAADF-STEM analysis was used to examine the differences in lithiation states between the central and surface primary particles in the fully calcined cathode. Fig. 2e and e1–e3 display three regions of interest (ROIs 1–3) from the center to the surface of a secondary particle in bare-NCM90. In ROI1, located near voids, smaller primary particles were found to contain an unlithiated rock salt phase (Fig. 2e1), as confirmed from the Fast Fourier Transform (FFT) image (Fig. 2e1) along the [100] axis. The magnified location and lattice structures are also presented in Fig. S5a and b.† Limited lithium contact likely caused this incomplete lithiation. ROI 2 (Fig. 2e2), representing a primary particle of normal size, was located slightly away from the void. This particle exhibited a well-formed layered structure with minimal Li/Ni mixing. In ROI 3, corresponding to a surface primary particle, the lattice displayed a rock salt layer approximately 2 nm thick (Fig. 2e3 and S5c†), transitioning into a 4 nm thick severely mixed layered phase before forming a layered structure with reduced Li/Ni mixing deeper into the particle. These observations highlight significant structural non-uniformity across secondary particles in bare-NCM90, despite low overall Li/Ni mixing as indicated by XRD (Fig. 2c and d).

For 25W-NCM90, three comparable regions (ROIs 4–6, Fig. 2f) were analyzed. ROI 4, located at the center of the secondary particle, exhibited no rock salt phase, and the lattice spacing of the transition metal (TM) layer was approximately 0.475 nm, consistent with the layered oxide crystal. STEM images across ROI 4–6 consistently showed well-defined layered structures free of the rock salt phase, despite slightly greater overall Li/Ni mixing in 25W-NCM90, as suggested by XRD refinement (Fig. 2c and d). Notably, 25W-NCM90 exhibit greater Li/Ni mixing in both the bulk and surface regions compared to bare-NCM90 according to STEM images. ROI 6 (Fig. 2h and the magnified image in Fig. S5d†) revealed a thick surface layer of layered oxide structure with severe Li/Ni mixing, where no rock salt phase or spinel phase was observed. These findings confirm that the WO_3_ coating facilitates homogeneous and complete lithiation during calcination, extending from the particle center to the surface.

We hypothesize that precursor surface properties significantly influence early-stage solid-state synthesis, including particle lithiation, nucleation, and growth. This early-formed layered oxide phase tends to coarsen rapidly on the secondary particle surface, where lithium is readily available, forming a dense layer that hinders lithium diffusion into the center. While defect-rich grain boundaries may facilitate lithium diffusion, the grain growth on the surface cause insufficient lithium supply to the central regions, leading to incomplete lithiation and formation of voids near the centers of secondary particles, as confirmed by FIB-SEM (Fig. 2a). Previous *in situ* TEM studies have observed the formation of a denser layered phase on the particle surface at around 300 °C.^17,18^ The present study underscores the critical role of precursor surface properties in determining structural heterogeneity within secondary particles. WO_3_ coating effectively mitigates such heterogeneity, whereas simple heat treatment increases heterogeneity through the formation of reactive NCMO surfaces. The pre-transformed NCMO accelerates the growth of dense surface layers, restricting lithium diffusion to the particle center. Consequently, central primary particles remain inadequately lithiated, leading to a lithium-deficient disordered layered phase or rock salt phase and a significantly decreased *I*_(003)_/*I*_(104)_ peak intensity ratio.

**Fig. 3:**
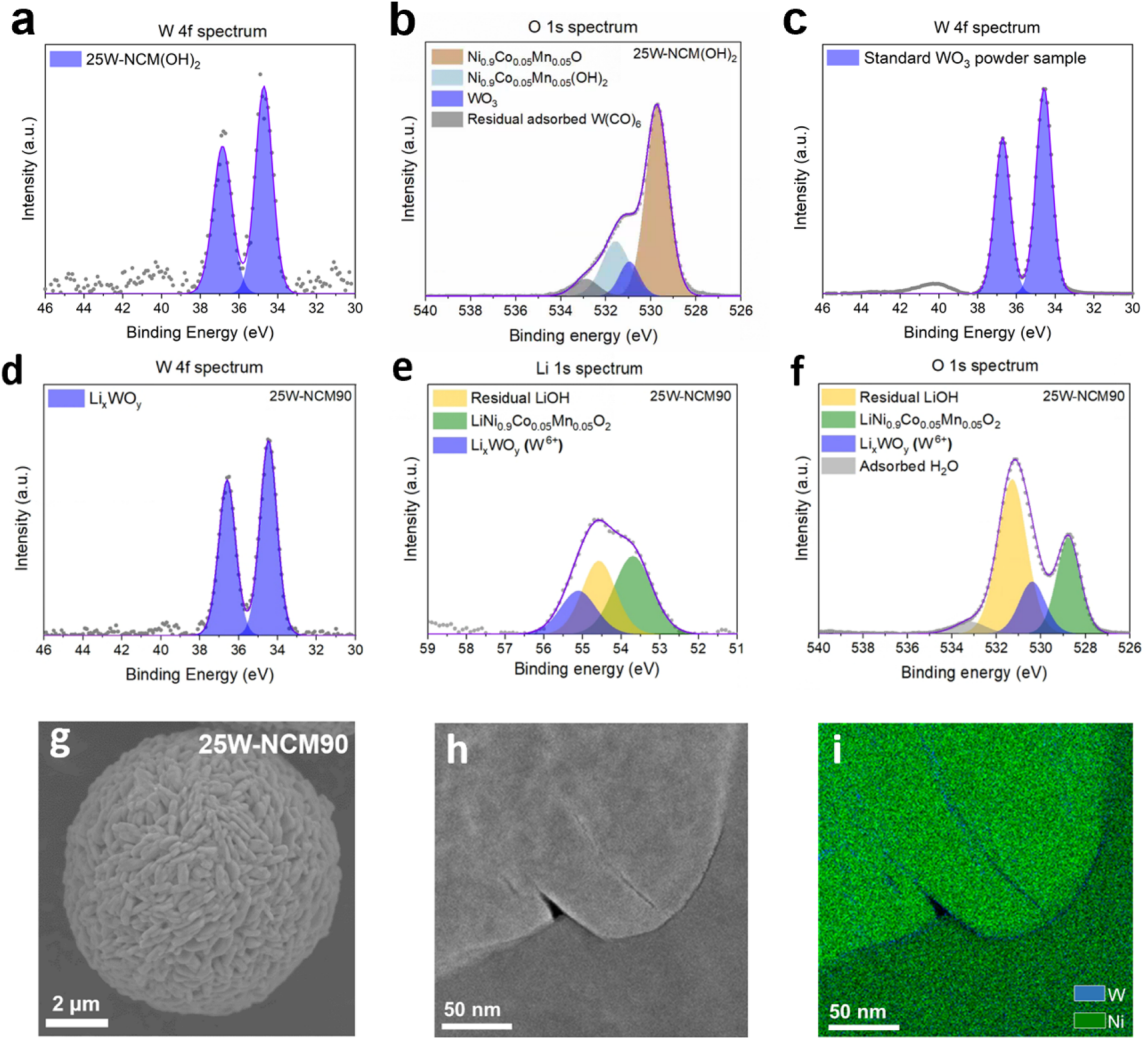
XPS spectra and TEM-EDS images identifying the distribution of W and transformation of W containing compounds in 25W-NCM90. (a and b) W 4f and O 1s spectra for 25W-NCM(OH)_2_. (c) W 4f spectra for commercial WO_3_ nano-powder. (d–f) W 4f, Li 1s and O 1s spectra for 25W-NCM90. (g) SEM image of a 25W-NCM90 secondary particle (h and i) TEM image and EDS elemental mapping of Ni and W along primary particle grain boundaries in 25W-NCM90.

To further investigate the role of the surface WO_3_ coating in the solid-state synthesis process, W-containing samples were characterized both before and after the high-temperature reaction. Using powder ALD coating, a uniform layer of WO_3_ was deposited on NCM(OH)_2_, achieving a linear growth rate of 0.11 mol‰ (W : TM + W) per cycle (Fig. S2†). XPS analysis was conducted for 25W-NCM(OH)_2_ (Fig. 3a and b) and 25W-NCM90 (Fig. 3d–f). The W 4f spectra of 25W-NCM(OH)_2_ exhibited two peaks at 34.7 eV and 36.8 eV (Fig. 3a), closely matching the characteristic peaks of commercial WO_3_ nano-powder at 34.6 eV and 36.7 eV (Fig. 3c), confirming that the ALD-deposited layer shares a similar chemical environment with WO_3_. This observation was further supported by the unique W–O peak at around 530.9 eV in the O 1s spectra of 25W-NCM(OH)_2_ and 25W-NCM (Fig. 3b), which was absent in the O 1s spectrum of h-NCM(OH)_2_ (Fig. S3a†). After the solid-state reaction with LiOH·H_2_O in O_2_, the W 4f peaks shifted slightly to lower binding energies (34.5 eV and 36.7 eV), confirming that W remained in the +6 oxidation state. Additionally, new peaks appeared at 530.8 eV in the O 1s spectra and 55.1 eV in the Li 1s spectra, consistent with the value reported for lithium tungsten oxide (LWO).^29^ Importantly, XPS, a surface sensitive characterization technique, revealed that the W/Ni peak intensity ratio (Ni 2p spectra of 25W-NCM(OH)_2_ and 25W-NCM90 are shown in Fig. S3b and c†) did not change significantly after the solid-state reaction. This suggests that the majority of tungsten atoms remain on the surface and do not diffuse into the NCM lattice to form dopants, even under high temperature calcination.

To corroborate these findings, TEM-EDS was used to analyze the spatial distribution of tungsten. Element mapping (Fig. 3h and i) of a grain boundary near the center of a 25W-NCM90 secondary particle showed that nickel (green color) was uniformly distributed, while tungsten (blue color) was concentrated at the grain boundaries rather than within the particle interiors. This observation aligns with the XPS results, confirming the poor solubility of tungsten in the NCM90 lattice. Previous reports also suggest that tungsten tends to form a surface coating rather than bulk doping.^30–32^ In the present study, we find that ALD-deposited WO_3_ was found to remain predominantly as a surface oxide, transforming into LWO during the solid-state reaction. Additionally, SEM-EDS analysis confirmed a uniform W distribution across the secondary particle (Fig. S4c–e†). However, due to the resolution limits of SEM and detection depth of EDS, it was not possible to differentiate whether W remained as a coating or had dissolved as a dopant in 25W-NCM90. Together, the XPS and TEM-EDS results demonstrate that ALD WO_3_ coating produces a tungsten-related surface layer that persists through the high temperature solid-state reaction. This surface layer is crucial in preventing the coarsening of primary particles during lithiation by forming a stable and insoluble coating. This mechanism preserves grain boundary integrity, enhances lithium-ion diffusivity along grain boundaries, and prevents excessive particle coarsening, particularly at relatively low temperatures where lithium diffusion within the bulk layered oxide crystal is slow.

**Fig. 4:**
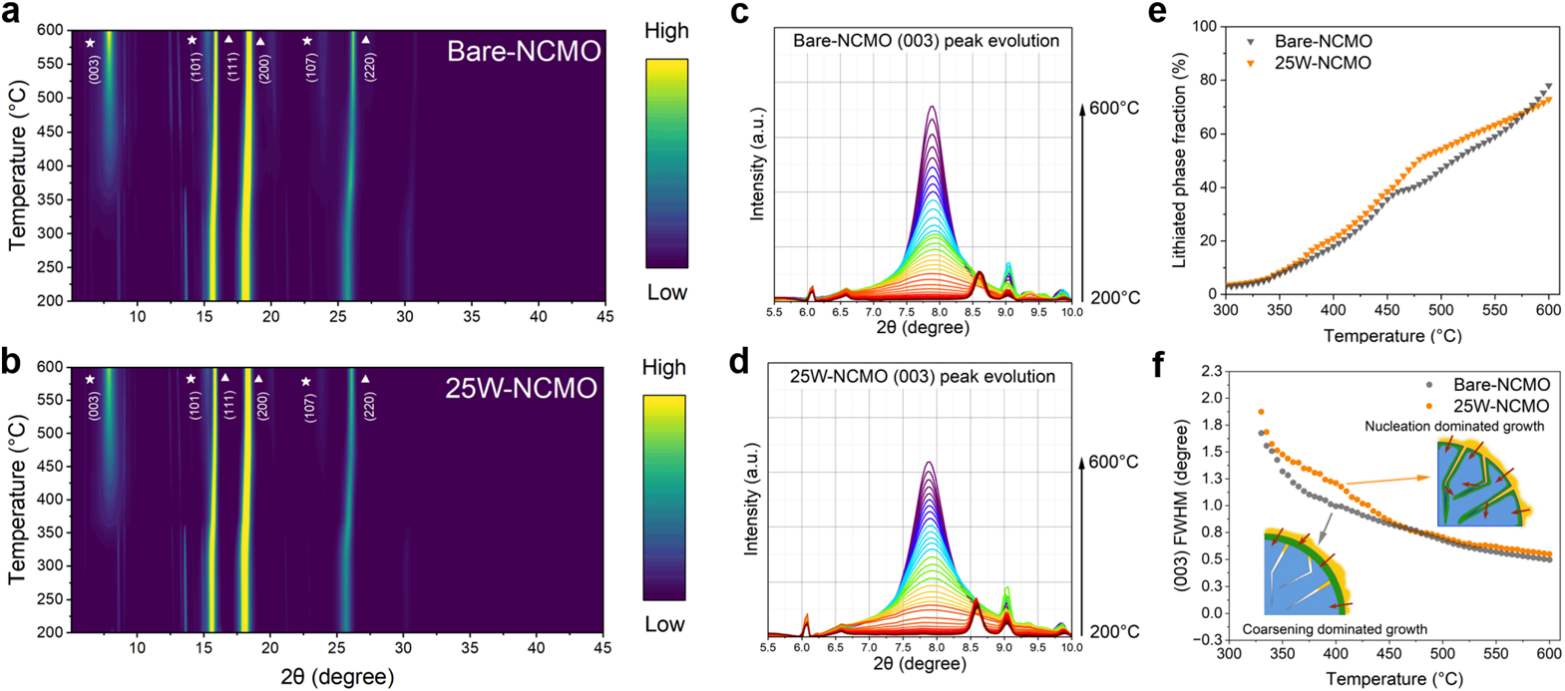
*Operando* synchrotron HT-XRD characterization of bare-NCMO and 25W-NCMO. (a and b) Contour plots showing XRD pattern evolution for bare-NCMO and 25W-NCMO mixed with LiOH·H_2_O during heating from 200 °C to 600 °C with a temperature ramping rate of 2 °C min^−1^. ★ indicates the layered phase and ▲ indicates the rock salt phase. (c and d) Evolution of the (003) peak from 200 °C to 600 °C. (e and f) Rietveld refinement results showing lithiated phase fraction growth and FWHM of the (003) peak for bare-NCMO and 25W-NCMO, respectively.

To comprehensively understand the role of the surface WO_3_ coating in the non-equilibrium solid-state reaction, *operando* HTXRD was used to monitor the evolution of lithiated phases.^14,19,33^ To minimize the influence from the (001) peak of NCM(OH)_2_ on the (003) peak of the layered oxide phase, NCM(OH)_2_ precursors were fully dehydrated at 350 °C for 3 h in an O_2_ atmosphere to form NCMO. The heat treatment did not significantly alter the morphology of the precursors (see Fig. S4a and b†) but likely increased surface reactivity, potentially enhancing structural non-uniformity in intermediates. The bare-NCMO and ALD-coated 25W-NCMO were mixed with LiOH·H_2_O in a molar ratio of 1 : 1.08 (TM : Li). Contour plots of *in situ* HTXRD data for bare-NCMO and 25W-NCMO, recorded between 200 °C and 600 °C, are shown in Fig. 4a and b. No significant phase changes were observed below 200 °C (Fig. S6†), apart from the gradual disappearance of LiOH·H_2_O crystalline peaks due to dehydration. To analyze lithiation kinetics during early-stage calcination, the evolution of the (003) peak for the layered oxide was examined (Fig. 4c and d). The average sizes of lithiated grains were compared using the full width at half maximum (FWHM) of the (003) peak and the Debye–Scherrer equation. As shown in Fig. 4f, the FWHM values for 25W-NCMO and bare-NCMO diverged at low temperatures, where nucleation and growth of the layered phase began. This divergence suggests that the WO_3_ surface coating may delay nucleation, suppress grain growth, or both.

To gain further insights, Rietveld refinement was performed on the XRD pattern to extract the fraction of the layered phase. The reaction mixture contained two primary crystalline phases: the NCMO rock salt phase and the Li_1−*x*_(NCM)_1+*x*_O_2_ layered oxide phase, where extra TM atoms occupy Li sites. Previous reports identified this lithiated phase as the initial reaction product.^13^Fig. 4e shows the relative phase fraction of the lithiated phase, calculated as the product of the phase fraction of Li_1−*x*_ (NCM) _1+*x*_ O_2_ and (1 − *x*). Below 450 °C, the relative phase fraction of Li_1−*x*_ (NCM) _1+*x*_ O_2_ was slightly higher in 25W-NCMO compared to bare-NCMO, while the FWHM of the (003) peak was larger for 25W-NCMO when at the same temperature. This suggests that the number of lithiated particles or nucleation sites for the layered oxide phase in 25W-NCMO is more than that of bare-NCMO during this period. These results indicate that lithiation kinetics in bare-NCMO are dominated by crystal growth and coarsening, where lithium primarily diffuses through the bulk of lithiated particles, leading to deeper lithiation. This bulk diffusion is hindered by the formation of dense layered phase surfaces during particle coarsening and grain boundary closure. In contrast, for 25W-NCMO, the ALD-deposited WO_3_ surface layer acts as an insoluble barrier, segregating lithiated particles at grain boundaries and preserving the grain boundary structure. A higher atom diffusivity has been previously reported for defect-rich grain boundaries than bulk.^34,35^ As lithium diffuses along grain boundaries, fresh NCMO surfaces within secondary particles begin lithiation, creating additional diffusion routes and nucleation sites. This mechanism enhances the lithiation uniformity of secondary particles.

To further quantify these findings, the initial lithiated particles are assumed to be approximately spherical for calculation purposes. The average volume of lithiated particles was calculated using the particle diameter along the (003) plane derived using the Debye–Scherrer equation (Fig. S8a†). The relative number of lithiated particles was then obtained by dividing the fraction of the lithiated phase by the average lithiated particle volume (Fig. S8b†). In bare-NCMO, the relative number of lithiated particles consistently decreased as temperature and the fraction of the lithiated phase increased, confirming that its lithiation kinetics are dominated by crystal growth and coarsening. Conversely, in 25W-NCMO, the number of lithiated particles increased between 350 °C and 400 °C after an initial period of coarsening. This supports the hypothesis that the WO_3_ surface layer promotes lithiation uniformity by maintaining the grain boundary structure and introducing new nucleation sites.

**Fig. 5:**
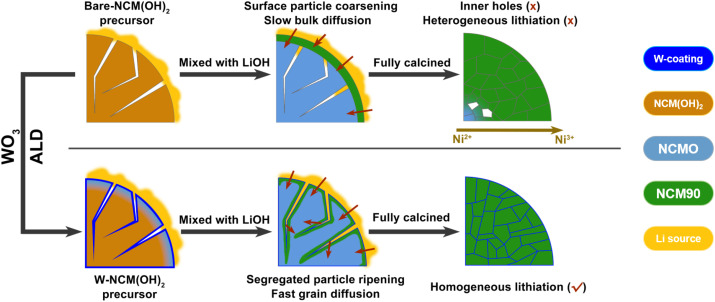
Schematics showing the reaction processes for bare and WO_3_-coated precursor particles during the high temperature solid-state reaction. The bare hydroxide precursor undergoes coarsening of surface-lithiated particles during early calcination, closing surface grain boundaries and suppressing further lithium diffusion. In contrast, the WO_3_ coating preserves the grain boundary structure, leading to a homogeneous lithiation, resulting in fully calcined particles with a uniform structure.

Fig. 5 illustrates the solid-state lithiation kinetics of bare and WO_3_ coated precursors, highlighting the effects of differing surface properties. For the non-coated pristine precursor, lithiation begins at around 300 °C with the formation of layered phase particles on the surface. As the temperature increases, these particles coalesce, leading to crystal growth. During this process, surface grain boundaries merge, forming a dense layer that impedes lithium diffusion into the inner rock salt particles. Consequently, the lack of sufficient lithium supply results in the formation of poorly lithiated or unlithiated primary particles in the center of secondary particles, which can induce lattice distortions and internal stress. These effects may contribute to lower initial capacity and reduced cycle stability. In contrast, the WO_3_-coated precursor exhibits markedly different lithiation behavior due to the stability of the surface coating. The WO_3_ layer creates a significant energy barrier to particle coarsening, preserving the grain boundary structure and facilitating fast lithium transport to the inner precursor particles. This coating promotes nucleation not only on the surface of secondary particles but also along internal grain boundaries, enabling a homogeneous topotactic phase transformation from the surface to center. As a result, lithiation uniformity is enhanced, ensuring more consistent and efficient lithiation throughout the material.

**Fig. 6:**
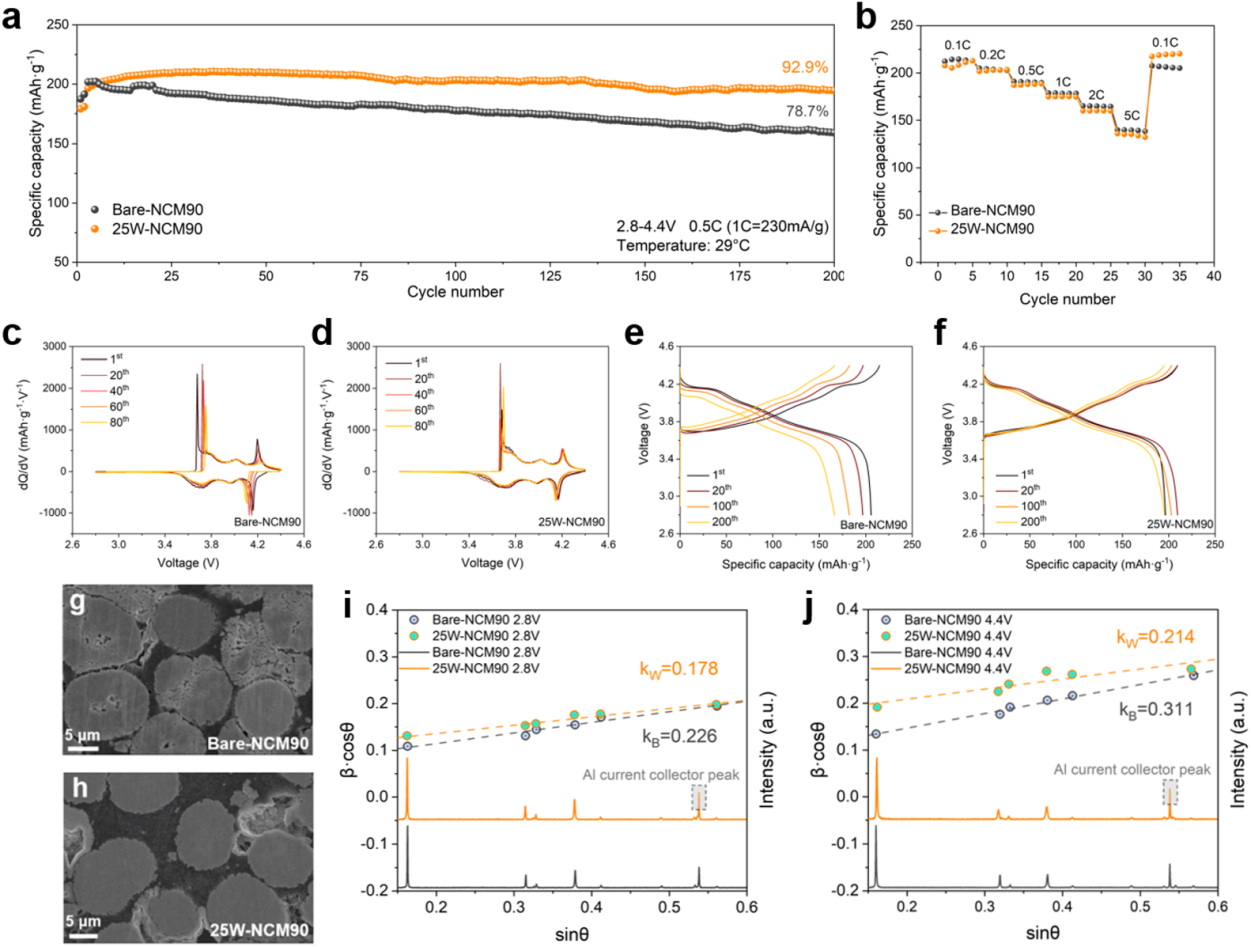
Electrochemical performance, cross-sectional SEM images, and strain behaviors of bare-NCM90 and 25W-NCM90. (a) Cycling stability of bare-NCM90 and 25W-NCM90 between 2.8 and 4.4 V at 0.5C (1C = 230 mA g^−1^). (b) Rate capability of bare-NCM90 and 25W-NCM90 from 0.1C to 5C. (c and d) Charge/discharge curves of bare-NCM90 and 25W-NCM90 between 2.8 V and 4.4 V. (e and f) Differential capacity curves of bare-NCM90 and 25W-NCM90. (g and h) Cross-sectional images of bare-NCM90 and 25W-NCM90 after 100 cycles at 1C. (i and j) XRD profiles of bare-NCM90 and 25W-NCM90 after discharging to 2.8 V(i) and charging to 4.4 V (j), along with Williamson–Hall plots.

The cycle performance of bare-NCM90 and 25W-NCM90 cathodes within a voltage range of 2.8–4.4 V (*vs.* Li/Li^+^) at a rate of 0.5C (1C = 230 mA g^−1^) was assessed (Fig. 6a). After two activation cycles at 0.1C, bare-NCM90 delivered an initial discharge capacity of 204.8 mA h g^−1^, retaining 78.7% capacity after 200 charge/discharge cycles. In contrast, 25W-NCM90 exhibited a slightly longer activation period, reaching its maximum discharge capacity of 210.0 mA h g^−1^ after 25 cycles, and showed superior capacity retention of 92.9% after 200 cycles. The higher discharge capacity confirms its greater electrochemically active lithiated phase compared to bare-NCM90. The rate capability test (Fig. 6b and S7†) further underscores the importance of precisely controlling the thickness of the surface coating using ALD. The charging/discharging profiles of bare-NCM90 and 25W-NCM90 (Fig. 6c and d) show that 25W-NCM90 experiences less capacity degradation and overpotential increase, indicating its superior surface stability during cycling. Differential capacity profiles for these two cathodes are presented in Fig. 6e and f. During the charging process, NCM cathodes typically undergo the sequential phase transformation H1 → M → H2 → H3, as lithium ions are extracted from the lattice structure, as labeled in the curve.^36,37^ The H2 to H3 transformation occurs when the remaining lithium ion in the layered structure is less than 25%, during which period the layered structure is prone to degradation into an electrochemically inactive rock salt phase. 25W-NCM90 exhibits good reversibility of the H2 to H3 transformation, which serves as a key indicator of structural stability. Conversely, bare-NCM90 exhibits a larger decline in the intensity of this transformation peak, highlighting its higher susceptibility to structural degradation.

Fig. 6g and h compare the cross-sectional SEM images of bare-NCM90 and 25W-NCM90 cathodes after charge/discharge cycling at 1C for 100 cycles, followed by a half-cycle charge to 4.4 V. Bare-NCM shows severe structural degradation of secondary particles, with cracks primarily originating at the particle centers. In comparison, 25W-NCM90 shows fewer internal cracks, maintaining its structural integrity. This superior capacity retention of 25W-NCM90 results from the stable LWO surface coating, which reduces further side reactions at the grain surface–electrolyte interface, and the radially aligned rod-like particles formed by the introduction of the high-valence element, which suppresses crack formation, as shown in Fig. 2b.^31,38–42^ To further evaluate the structural stability, Williamson–Hall plot (WHP) analysis was performed for bare-NCM90 and 25W-NCM90 to monitor their lattice strain behaviors during charge/discharge. After two activation cycles, XRD patterns and WHP profiles of both samples were obtained at their first discharge to 2.8 V and first charge to 4.4 V (Fig. 6i and j) to examine crystal lattice strain using the Williamson–Hall equation:^43,44^1
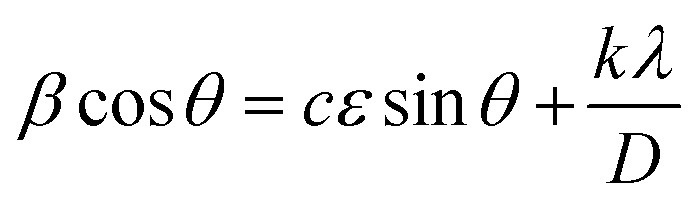
where *β* is the FWHM in radians, *θ* is the Bragg diffraction angle in radians, *λ* is the X-ray wavelength, *D* is the crystallite size, *c* and *k* are both constant for the same material and *ε* is the crystal lattice strain. For bare-NCM90 and 25W-NCM90, the constant *c* is assumed to be equal. As plotted in Fig. 6i and j, the slope of the *β* cos *θ versus* sin *θ* plot is proportional to crystal lattice strain. At the fully lithiated stage (2.8 V), 25W-NCM90 exhibited a lower lattice strain (*k*_W_, 0.178) compared to bare-NCM90 (*k*_B_, 0.226). Additionally, upon delithiation (4.4 V), the lattice strain of bare-NCM90 increased significantly by 37% to 0.311, whereas 25W-NCM90 showed a more moderate increase of 20%, reaching 0.214. This milder strain behavior in 25W-NCM90 preserves secondary particle structural integrity, preventing crack formation during delithiation.^45^ The initial strain caused by lattice distortion was also analyzed by geometry phase analysis (GPA), as shown in Fig. S9.† Bare-NCM90 shows a significantly larger and more uneven strain distribution along the [003] direction than 25W-NCM90, consistent with the WHP results in Fig. 6i. This uneven strain likely originated from mismatched rock salt phases at particle centers. These findings confirm the electrochemical stability provided by the LWO coating and its role in maintaining a uniform and robust structure.

## Conclusion

This study investigated the lithiation mechanism of polycrystalline hydroxide precursors during high temperature solid-state synthesis. It was found that the early-stage formation and coalescence of layered phase primary particles on secondary surfaces suppressed lithium diffusion, causing structural non-uniformity and poor electrochemical performance. To address this, a stable, insoluble surface layer was perceived. Using powder ALD, a WO_3_ layer was deposited uniformly on polycrystalline NCM(OH)_2_ precursors. This layer transformed *in situ* into LWO during high temperature solid-state synthesis, acting as a segregation layer. Characterization *via* ICP-OES, XPS, and TEM-EDS confirmed the low solubility of tungsten in the NCM lattice and the stability of the LWO layer. *Operando* HTXRD analyses elucidated the surface-dependent nucleation and growth mechanisms, revealing that the modification preserved high-diffusivity grain boundaries and facilitated uniform lithiation into the central regions of secondary particles. This prevented the formation of inner voids and residual rock salt phases, as confirmed by SEM and TEM. The modification enhanced both the structural stability of secondary particles and surface electrochemical stability of primary particles. This work advances the understanding of early-stage solid-state reactions and provides a pathway to achieve homogeneity in high temperature solid-state reactions for next-generation cathode materials.

## Experimental

### Materials synthesis

The spherical Ni-rich Ni_0.9_Co_0.05_Mn_0.05_(OH)_2_ precursor was purchased from CNGR Advanced Material Co. Ltd. ALD of tungsten oxide on the Ni_0.9_Co_0.05_Mn_0.05_(OH)_2_ precursor was performed in a powder ALD system (GT10 powder ALD, Yunmao) integrated with an Ar-filled glove box using tungsten hexacarbonyl (W(CO)_6_) (99.9%, Macklin) and ozone (O_3_) as precursors at 200 °C. The source temperature for W(CO)_6_ was 75 °C, with a pulse time of 4 s to achieve a partial pressure of ∼0.1 Torr. The pulse time for O_3_ is 1.2 s under a mass flow of 100 sccm. Each pulse was separated by a 60 s Ar purge. The Ni_0.9_Co_0.05_Mn_0.05_(OH)_2_ precursor treated with *n* cycles of WO_3_ ALD was labeled as *n*W-NCM(OH)_2_. Bare-NCM and *n*W-NCM cathode materials were prepared by mixing Ni_0.9_Co_0.05_Mn_0.05_(OH)_2_ or *n*W-NCM(OH)_2_ with LiOH·H_2_O (Aladdin) in a molar ratio of Li : TM = 1 : 1.08, followed by calcination in a tube furnace (Lindberg/Blue Mini-Mite, Thermo fisher) under a pure oxygen atmosphere with a 40 sccm oxygen flow.

### Material characterization

The chemical composition and the amount of ALD coated WO_3_ were measured by ICP-OES using an Icap7400 ICP-OES Analyzer (Thermo Fisher). For W measurement, the powder was soaked in 20% NaOH solution and diluted, as WO_3_ has a poor solubility in conventional acids. For Ni, Co, Mn measurements, the bare-NCM(OH)_2_ and bare-NCM powder was dissolved in aqua regia and diluted. Notably, both *n*W-NCM(OH)_2_ and *n*W-NCM90 (*n* ≥ 25) have poor solubility in aqua regia or HF solution so the amount of Ni, Co and Mn cannot be accurately measured. X-ray photoelectron spectroscopy data were collected on an ESCALAB 250Xi (Thermo fisher) using an Al Kα (*hν* = 1486.7 eV) X-ray source. All *ex situ* X-ray diffraction patterns were collected on a D8 advance (Bruker) with Cu Kα radiation and a 0.02° step size. *Operando* HTXRD data were collected at the Shanghai Synchrotron Radiation Facility (SSRF) on beamline BL02U2. Rietveld refinements of the collected XRD patterns were conducted using the GSAS II package. Morphologies and cross-sectional images of the prepared particles were obtained by scanning electron microscopy (JSM-7800F and JSM-6010PLUS/LA, JEOL). The cross-sections of the cycled cathode were prepared using cross-section polisher IB-19520CCP (JEOL). Transmission electron microscopy samples were prepared *via* a focused-ion beam system (JIB-4700F, JEOL), and scanning transmission electron microscopy (STEM) images were collected on a Grand ARM-300F (JEOL). Lattice distortion mapping was performed using Strain++ (https://www.jjppeters.github.io/Strainpp/).^46^

### Electrochemical measurements

The cathode slurry was prepared by mixing NCM90 powder, Super P and polyvinylidene fluoride (PVDF) binder in a weight ratio of 8 : 1 : 1 in *N*-methyl pyrrolidine (NMP) using a THINKY MIXER. The slurry was cast onto Al current collector foil and dried in an N_2_ atmosphere at 80 °C for 30 min, following by vacuum drying at 80 °C for 12 h. The final active material loading was 3–4 mg cm^−2^. Electrochemical measurements were conducted in R2032 coin cells. The electrolyte consisted of 1.2 M LiPF_6_ dissolved in ethyl carbonate (EC) and ethyl methyl carbonate (EMC) (3 : 7 by volume) with 2 wt% vinylene carbonate (VC) as an additive. Battery testing was performed using a Neware BTS4000-5 V battery tester in a constant temperature environment of 29 °C.

## Data availability

The data supporting this article have been included as part of the ESI.†

## Author contributions

Y. W., X. C. and J. X. conceived the idea and designed experiments in detail. Y. W. carried out the material fabrication, electrochemical measurements, and data analysis and wrote the manuscript. Y. W. and X. C. carried out the SEM characterization, *in situ* HTXRD experiment, Rietveld refinements and FIB sample preparation. W. L. and Y. D. carried out the XPS experiments. W. L. carried out the TG analysis. Q. Z. and P. Y. carried out the TEM characterization and helped with TEM data analysis. Y. W., H. L. and G. Z. carried out the ICP-OES experiments. J. X. supervised the experiment and revised the manuscript.

## Conflicts of interest

The authors declare that they have no known competing financial interests or personal relationships that could have appeared to influence the work reported in this paper.

## Supplementary Material

SC-OLF-D5SC00271K-s001
