# Supplementary material for: Enabling uniform lithiation in solid-state synthesis by preventing pre-matured surface grain coarsening through grain boundary engineering
Source: Chem Sci. 2025 Apr 25;16(22):9809–19. doi: 10.1039/d5sc00271k (PMC12042692; doi:10.1039/d5sc00271k)
Supplement: SC-016-D5SC00271K-s001 [file SC-016-D5SC00271K-s001.pdf]

Supplementary Information (SI) for Chemical Science.
This journal is © The Royal Society of Chemistry 2025

Supplementary Information

Enabling uniform lithiation in solid-state synthesis by preventing pre-matured surface grain coarsening through grain boundary engineering

Yifan Wu,[a] Xincan Cai,[a] Weiyi Lin,[a] Yingdong Deng,[a] Qing Zhang,[ab] Haoyuan Li,[a] Pu Yan,[a] Guohui Zhong,[a] and Jin Xie*[ab]

[a] *School of Physical Science and Technology, ShanghaiTech University, Shanghai 201210, China*

[b] *Shanghai Key Laboratory of High-resolution Electron Microscopy, ShanghaiTech University; Shanghai 201210, China*

* *Corresponding author – xiejin@shanghaitech.edu.cn*

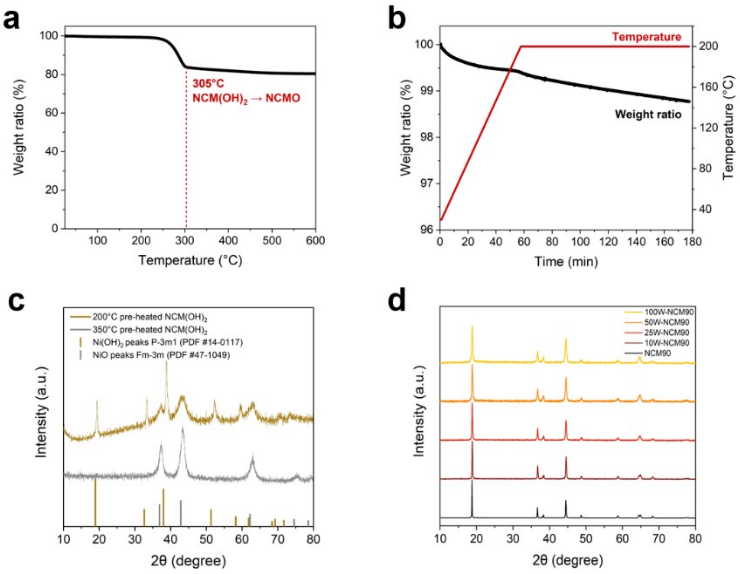

Figure S1.TGA and XRD analysis. (a) Bare-NCM$(OH)_2$ TGA curve from 25°C to 600°C at a ramping rate of 3°C/min. (b) Bare-NCM$(OH)_2$ TGA curve from 25°C to 200°C at a ramping rate of 3°C/min and 200°C holding for 2h. Both of these two TGA experiment are performed in O_2 atmosphere. (c) XRD patterns of the Bare-NCM$(OH)_2$ at 200°C and 350°C holding for 3 hours. The precursor shows a mixture of hydroxide phase and rocksalt phase. (d) XRD patterns of the fully calcined WO_3-coated samples (10W-NCM90, 25W-NCM90, 50W-NCM90 and 100W-NCM90) at 750°C in O_2 with $LiOH \cdot H_2O$ for 12h. The $I_{(003)}/I_{(104)}$ ratio decreases as WO_3 coating amount increases.

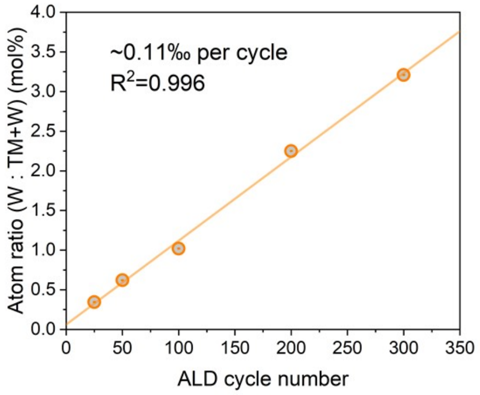

Figure S2. ALD deposited W molar amount of 25W-NCM$(OH)_2$, 50W-NCM$(OH)_2$, 100W-NCM$(OH)_2$, 200W-NCM$(OH)_2$, and 300W-NCM$(OH)_2$ obtained from ICP-OES. The deposition amount shows linear relationship to ALD cycle with an amount of about 0.11‰ molar ratio of tungsten to transition metal plus tungsten per cycle.

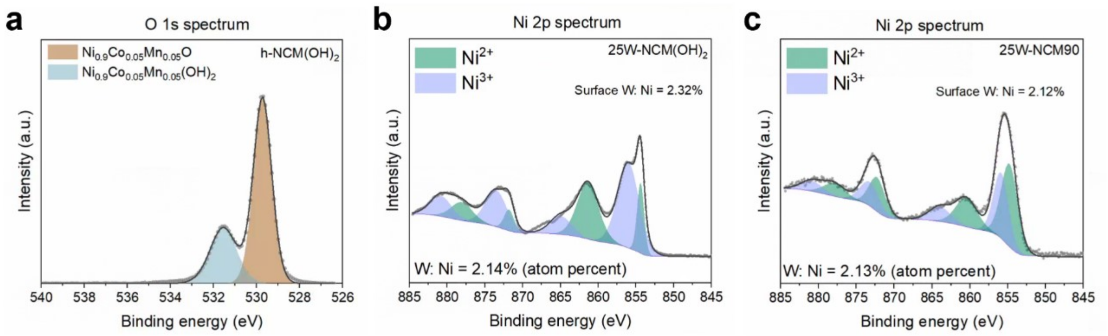

Figure S3: XPS spectra of h-NCM$(OH)_2$, 25W-NCM$(OH)_2$ and 25W-NCM90. (a) O 1s spectra for h-NCM$(OH)_2$, (b) Ni 2p spectra for 25W-NCM$(OH)_2$ and (c) Ni 2p spectra for 25W-NCM90. The W/Ni ratio are 2.32% and 2.12% for 25W-NCM$(OH)_2$ and 25W-NCM90 each, calculated from XPS spectra.

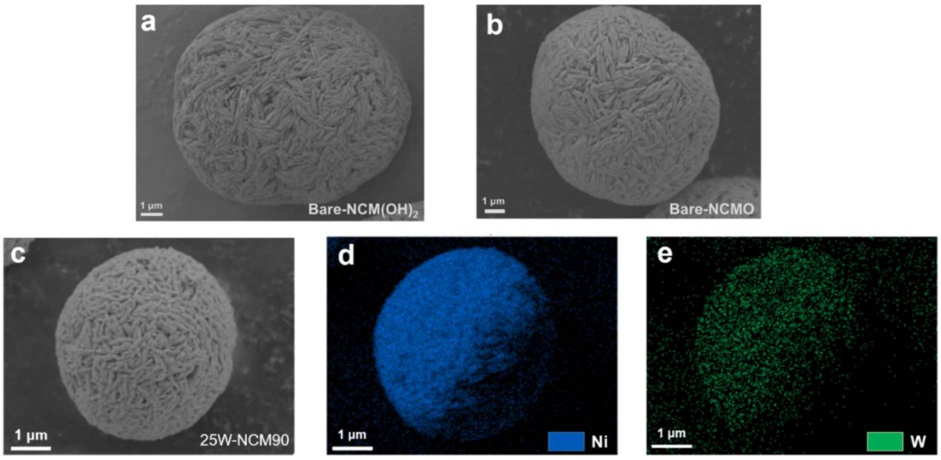

Figure S4. SEM images of (a) a Bare-NCM$(OH)_2$ secondary particle and (b) a Bare-NCMO secondary particle. (c-e): SEM-EDS mapping of a 25W-NCM90 particle. Deep blue and green represent Ni, W signal respectively. W is "uniformly distributed in primary particles" in SEM-EDS mode.

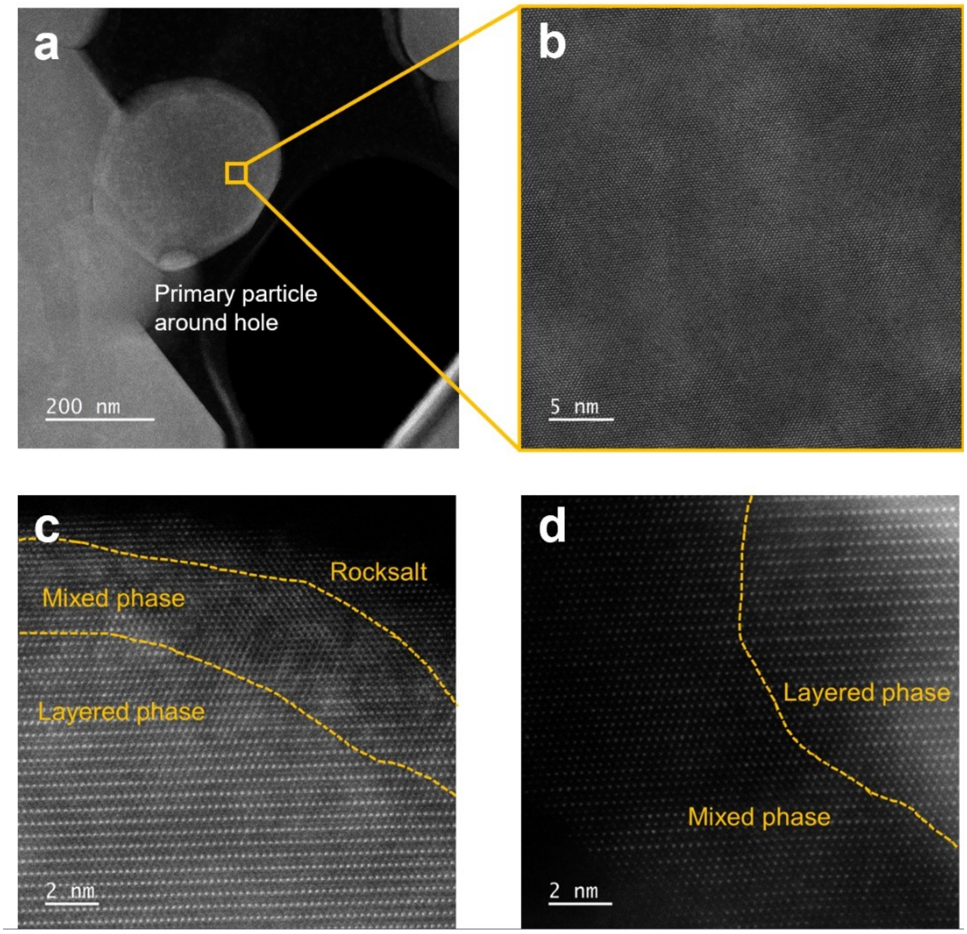

Figure S5. HADDF-STEM images for a center primary particle of Bare-NCM90 and the surface of a primary particle of Bare-NCM90 and 25W-NCM90 (a) Position of inner rock salt particle in Bare-NCM. (b) Magnified lattice structure of (a) shows a rock salt phase. (c) Lattice structure of Bare-NCM surface primary particles. (d) Lattice structure of 25W-NCM surface primary particles. All HAADF-STEM images were captured along the <100> zone axis in the $R\bar{3}m$ lattice.

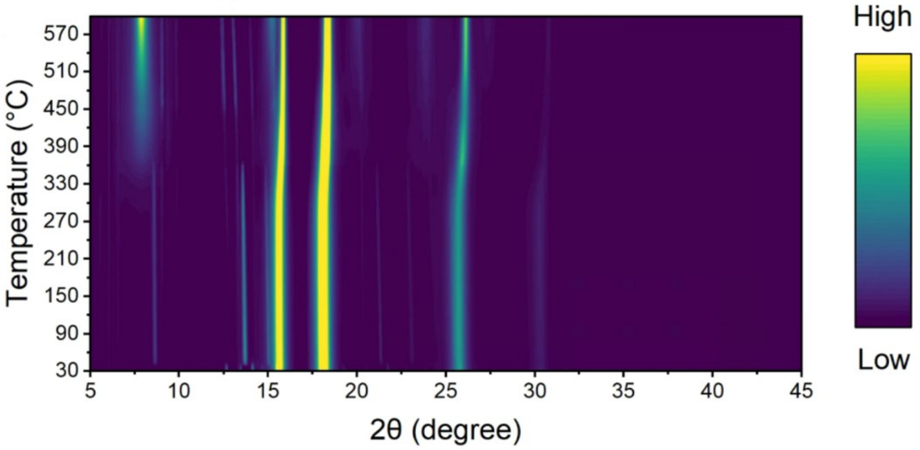

Figure S6. Contours plots of the *operando* HTXRD pattern from room temperature to 600°C with a temperature ramping rate of 2°C/min. No obvious change except for LiOH·H_2O dehydration are observed below 200°C

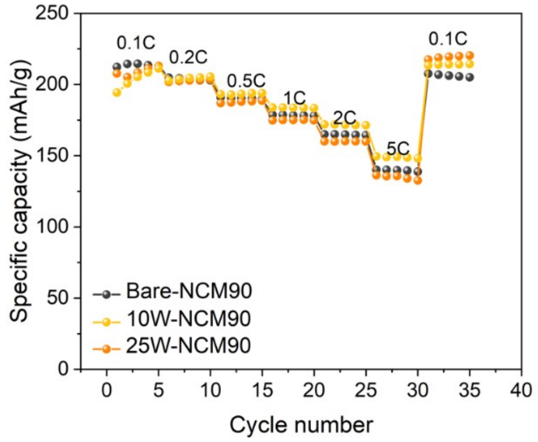

Figure S7: Rate capability of Bare-NCM90, 10W-NCM90 and 25W-NCM90 at 0.1C, 0.2C, 0.5C, 1C, 2C and 5C.

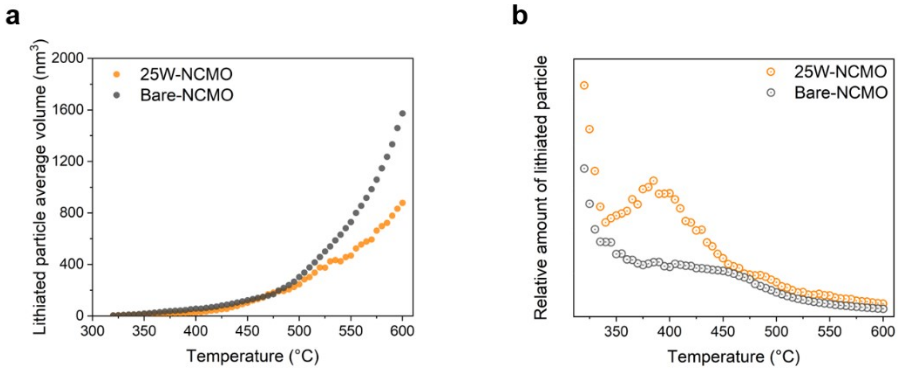

Figure S8: (a) The estimated average volume of lithiated particle and (b) relative amount of lithiated particle for Bare-NCMO and 25-NCMO during *operando* HTXRD calcination with $LiOH \cdot H_2O$.

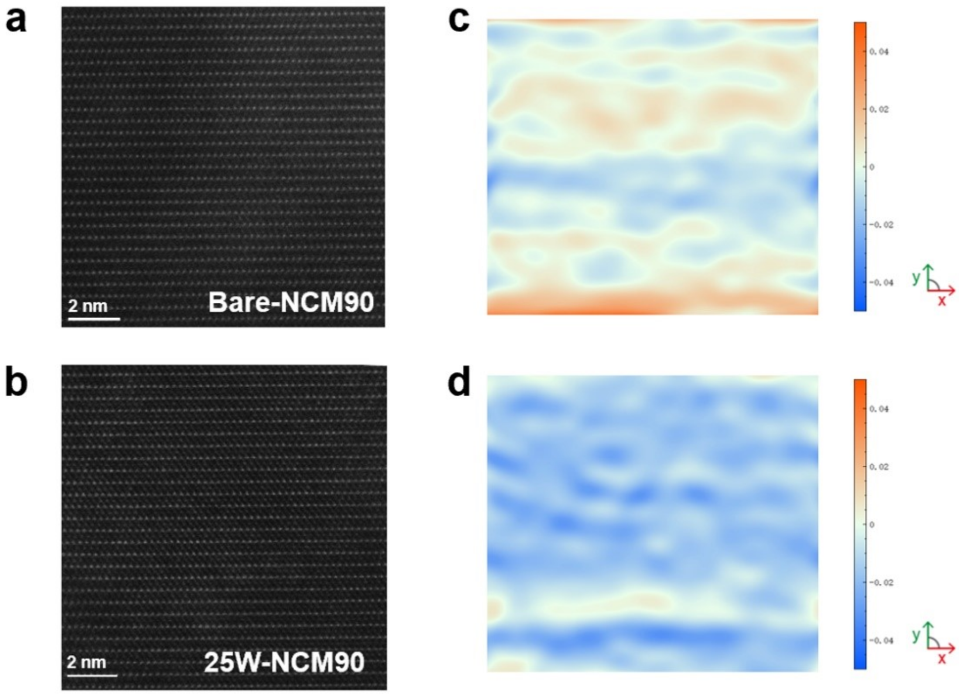

Figure S9: Geometry phase analysis (GPA) of Bare-NCM90 and 25W-NCM90. (a, b) HAADF-STEM image of a particle in Bare-NCM90 and 25W-NCM90. (c, d) Lattice distortion ε_e of Bare-NCM90 and 25W-NCM90 respectively. Red area represents lattice shrinkage and blue area represent stretching. All GPA results have cut off the top and bottom atoms layer to avoid calculation error at atom layer boundaries.

Table S1: Chemical composition of purchased $NCM(OH)_2$ hydroxide precursor determined by ICP-OES.

Chemical composition of $NCM(OH)_2$			
Elements	Ni	Co	Mn
Concentration (ppm)	580.706	32.859	30.345
Atoms percentage (%)	89.91%	5.07%	5.02%
